# Soybean *β*-Conglycinin Inhibits Broiler Growth and Nutrient Utilization by Inducing Allergic and Inflammatory Responses, Impairing Intestinal Barrier Integrity and Altering Cecal Microbiota

**DOI:** 10.3390/ani15121701

**Published:** 2025-06-09

**Authors:** Yusong Du, Zixi Yu, Shasha Wan, Yunfei Li, Rujie Liu, Jiaxuan Zhang, Zewei Sun, Qingzhen Zhong

**Affiliations:** Jilin Province Key Laboratory of Animal Nutrition and Feed Science, College of Animal Science and Technology, Jilin Agricultural University, Changchun 130118, China; 18904468337@163.com (Y.D.); yzx010921@outlook.com (Z.Y.); 19327901147@163.com (S.W.); 18843181003@163.com (Y.L.); rujieliu@126.com (R.L.); jasonchang0817@outlook.com (J.Z.)

**Keywords:** *β*-conglycinin, broiler nutrition, growth, anti-nutritional factors

## Abstract

This study aimed to comprehensively investigate the impact of soybean *β*-conglycinin on broiler growth performance, nutrient utilization, allergic and inflammatory responses, intestinal barrier integrity, and cecal microbiota. A total of 168 newly hatched (1-day-old) Arbor Acres broilers with similar body weights were randomly divided into 6 treatment groups with 4 replicates of 7 broilers per replicate: the control group received a soybean-free basal diet, while the remaining five experimental groups were provided with diets supplemented with purified soybean *β*-conglycinin at the doses of 1%, 2%, 3%, 4%, and 5%. The results demonstrated that dietary 1–5% *β*-conglycinin supplementation significantly reduced the growth performance, nutrient utilization, and content of digestive enzymes in broilers (*p* < 0.05). Dietary 1–5% *β*-conglycinin supplementation also significantly increased the serum levels of histamine, *β*-conglycinin-specific IgY and IgM, TNF-α, and IL-6 and decreased IL-10 levels; the 3% group had the highest levels of histamine, TNF-α, and IL-6 and the lowest levels of IL-10 (*p* < 0.05). *β*-conglycinin supplementation significantly down-regulated the mRNA expression of tight junction proteins, *MUC2* and *IL-10*, and up-regulated the expression of *TNF-α* and *IL-6* in the small intestinal mucosa (*p* < 0.05). Furthermore, the Shannon and Simpson indices were significantly reduced by dietary 1–5% *β*-conglycinin administration (*p* < 0.05). The relative abundance of beneficial bacteria (*Blautia*, *Lactobacillus*, and *Butyricoccus*) was significantly decreased in all treatments (*p* < 0.05). Taken together, these findings suggest that *β*-conglycinin induces allergic and inflammatory responses, impairs intestinal barrier integrity, and alters the intestinal microbial balance, ultimately leading to reduced nutrient utilization and inhibited growth in broilers. Notably, our study demonstrated that dietary supplementation with 1% *β*-conglycinin already had various negative effects on broilers, and particularly supplemental 3% *β*-conglycinin induced serious allergic and inflammatory reactions. Therefore, in the present study, it is recommended that the inclusion level of *β*-conglycinin in broiler formula feed should not exceed 1%, i.e., the *β*-conglycinin content in the diet should not exceed 0.6% (converted from *β*-conglycinin purification purity).

## Simple Summary

Soybeans are widely used in poultry production due to their balanced amino acid composition and high protein content. However, soybeans have been considered as one of the eight major allergenic foods. *β*-conglycinin has been identified as one of the two major antigen proteins in soybeans that induce allergic reactions in humans and animals. Therefore, this study aimed to investigate whether varying doses of *β*-conglycinin elicit a negative effect on broilers. The results showed that the dietary inclusion of 1%, 2%, 3%, 4%, and 5% *β*-conglycinin induced allergic and inflammatory responses, compromised intestinal barrier integrity, and disrupted the balance of the intestinal microbiota, thereby exerting inhibitory effects on nutrient utilization and growth performance in broilers. This will serve as a valuable reference for the appropriate level of soybeans and their products included in the formula of broilers and contribute to the scientific allocation of soy protein sources in poultry production.

## 1. Introduction

Soybeans are one of the major vegetable protein sources, possessing high nutritional value and being rich in protein as well as numerous essential amino acids. Therefore, soybeans and soybean meal have become crucial sources of dietary protein for poultry and livestock. However, soybeans are one of the top eight food allergens, which pose a serious threat to the health of humans and various animals [1,2]. Several studies have documented the presence of various anti-nutritional factors (ANFs) in soybeans, particularly the antigenic proteins that elicit allergic reactions in young animals [3]. *β*-conglycinin, also known as the 7S protein, is one of the major soybean antigenic proteins. It is highly immunogenic and constitutes 30–35% of the total seed protein. *β*-conglycinin is a trimer glycoprotein composed of three subunits that bind to each other through hydrophobic interactions, namely the *α*′ (76 kDa), *α* (72 kDa), and *β* (52 kDa) subunits [4], and *β*-conglycinin contains 14.5% *α*-helix, 42% *β*-sheet, and 33.5% *β*-strand and random coils [5]. Furthermore, several studies have also demonstrated that all three subunits of *β*-conglycinin possess allergenic potential [6].

Extensive research has shown varying degrees of negative effects of *β*-conglycinin on animals, which will limit the use of soybeans and soybean meal in animal production. Studies conducted on piglets, fish, calves, and mice have demonstrated that excessive *β*-conglycinin induced allergic reactions [7,8], triggered inflammatory responses in the intestine [9,10], disrupted intestinal barrier integrity [7,11], and altered microbial communities [12,13], consequently inhibiting growth [14,15].

In comparison to the extensive and in-depth research conducted on mammals and aquatic animals, research on soybean antigenic proteins for poultry is very limited. Currently, there are only a few reports available, and they are controversial. For instance, Kang et al. [16] demonstrated that feeding broilers with 60 mg *β*-conglycinin at seven days of age resulted in damage to the intestine and an increase in mast cell numbers within the intestine, as well as significant increases in the mRNA expression of inflammatory factors (TNF-*α*, IL-8, and IL-2). However, Osman et al. [17] found that the dietary supplementation of glycinin could improve growth performance while reducing abdominal fat in broilers. To the best of our knowledge, the immune and digestive systems of birds exhibit significant differences compared to those of other animals, providing a compelling rationale for further research on soybean antigenic proteins specifically for birds.

Therefore, this study aimed to investigate the impact of *β*-conglycinin on the growth performance, nutrient utilization, allergic and inflammatory reactions, intestinal barrier integrity, and cecal microbiota of broilers through dietary addition with varying levels of purified soybean *β*-conglycinin. The findings presented in this study will determine whether varying doses of *β*-conglycinin elicit a negative effect on broilers. This will serve as a valuable reference for the appropriate level of soybeans and their products included in the formula of broilers and contribute to the scientific allocation of soy protein source feeds in poultry production.

## 2. Materials and Methods

### 2.1. Purification of β-Conglycinin (7S Globulin)

Low-temperature defatted soybean powder (Yihai Kerry Arawana Holdings Co., Ltd., Shanghai, China) was used to extract purified *β*-conglycinin. Following the simplified ultrafiltration membrane method described by Wu et al. [18], a substantial quantity of extracted *β*-conglycinin (yield and purity of 19.7% and 62.6%) was utilized in broiler feeding experiments. The *β*-conglycinin extracted by the simplified ultrafiltration membrane method was determined to have a dry matter content of 98.04%, crude protein content of 92.1%, and ether extract content of 2.13%. For the determination of specific antibodies, high-purity *β*-conglycinin extraction (yield and purity of 5% and 98%) was carried out using the immunological methods proposed by Iwabuchi and Yamauchi [19]. Therefore, due to the limitation of extraction efficiency, the purified *β*-conglycinin was administered to 168 chickens over 21 days in this trial.

### 2.2. Bird Management and Experimental Diets

This study was conducted in accordance with the Animal Care requirements of the Animal Administration Committee at Jilin Agricultural University (Permit Number: SYXK(Ji)2023-0024). A total of 168 one-day-old Arbor Acres broilers (purchased from Jilin Dexiang Livestock Co., Ltd., Dehui, China) with similar initial body weight were randomly allocated to 6 groups with 4 replicates of 7 broilers per replicate, and the trial period was 21 days. The control group (CON) was on the soybean-free basal diet. The other five groups were supplemented with a diet containing 1.0%, 2.0%, 3.0%, 4.0%, and 5.0% of purified *β*-conglycinin. The supplemented level was referred to in the studies of Chen et al. [20] and Zhang et al. [21]. All six experimental diets were isonitrogenous and isoenergetic and were formulated in accordance with the Feeding Standard for Chickens (NY/T 33-2004) and NRC (1994) [22] feeding standards. The AME of *β*-conglycinin was calculated based on the determination of the nutrient content of *β*-conglycinin and a formula provided by Tian and Li [23]. The dietary composition and nutrient levels are detailed in Table 1. Cages and utensils were cleaned and fumigated with formaldehyde for 1 week before the experiment. The experiment employed continuous lighting for the first 3 d, and a lighting schedule of 16 h light and 8 h dark was provided during the surplus experimental period. The environmental temperature was initially set at 32 °C for the first 3 d, which was gradually reduced by 1 °C every three days until reaching a target temperature on d 21. The average air humidity was set at 55–65%. All broilers had free access to feed and clean water during the experiment.

### 2.3. Growth Performance Data Collection

The broilers were weighed on fasting weekly, and data on the initial body weight (IBW) and final body weight (FBW) of birds in each cage were recorded, and the total amount of feed in the feed tray and the amount of feed left over for the next day were recorded daily. The average daily gain (ADG), average daily feed intake (ADFI), and feed conversion ratio (FCR) were calculated from the recorded data to evaluate the growth performance of broilers.

### 2.4. Sample Collection

Before the feeding experiment began, the experimental diets were sampled once and stored at −20 °C for chemical analysis. On d 17, 18, 19, and 20, a metabolic test was carried out by using the full fecal collection method. Fresh excreta (free from contaminants, e.g., feathers, feed residues) were collected twice by wrapping the trays under each cage with a clean plastic sheet per day and were pooled by cage (replicate) and sprayed with 10 mL of 10% sulfuric acid solution per 100 g of excreta and kept at −20 °C until analysis. All excreta collected for 4 d were dried to constant weight at 65 °C and weighed after 24 h at room temperature to determine moisture loss.

On the 21st day at 8:00 a.m., 3 broilers from each replicate were randomly chosen to be stunned electrically, then immediately slaughtered via exsanguination, plucked, and sampled. Blood samples were collected from jugular veins and centrifuged (3000 rpm, 4 °C, 10 min); then the serum was collected and stored at −20 °C for analysis. The broilers were dissected to obtain the tissues of the duodenum, ileum, and jejunum, then flushed gently with cold saline solution, and the mucosal tissue was scraped with a sterile glass slide from the intestine after being washed with ice-cold saline solution and stored at −80 °C. Meanwhile, the contents of the duodenum and caecum were quickly collected on ice, snap-frozen in liquid nitrogen, and also stored at −80 °C for further analysis.

### 2.5. Nutrient Utilization

Dry matter (DM) content was obtained by drying at 105 °C to constant weight (Method 930.15). Crude protein contents were assayed using an automatic Kjeldahl nitrogen apparatus (LeiCi KDN-1, Shanghai, China) for nitrogen determination. Ether extract (EE) was determined after extraction with 99.5% diethyl ether in an accelerated extractor (Method 920.39). Crude ash (Ash) was incinerated at 550 °C in a muffle furnace and subsequently analyzed (Method 942.05). Crude fiber (CF) analysis involved treating the sample with a fixed amount of acid and base, followed by boiling under specified conditions. The ether extract was removed using ethyl ether and ethanol, and mineral content was eliminated through high-temperature combustion (Method 962.09). An adiabatic bomb calorimeter was used to calculate gross energy (GallenkampAutobomb, London, UK) standardized with benzoic acid [24]. All data were expressed on a DM basis and using the following formula:

Nutrient utilization rate % = (nutrient intake − nutrient voided)/(nutrient intake) × 100(1)

### 2.6. Analysis of Digestive Enzymes

The duodenal contents were homogenized in saline; the sample was centrifuged at 12,000 rpm, 4 °C; and the collected supernatant was stored at −80 °C until analysis. Chymotrypsin, amylase, lipase, and total protein (bicinchoninic acid method) in duodenal contents were measured with commercial assay kits (Suzhou Grace Biotechnology Co., Ltd., Suzhou, China).

### 2.7. Enzyme-Linked Immunosorbent Assay

According to the manufacturer’s instructions, the histamine, TNF-*α*, IL-6, and IL-10 levels in the serum were measured using chicken ELISA kits (Jiangsu Meibiao Biotechnology Co., Ltd., Nanjing, China). The DAO and D-LA in the serum were determined using ELISA kits (Suzhou Grace Biotechnology Co., Ltd., Suzhou, China).

An indirect ELISA was used to quantify IgY and IgM against *β*-conglycinin in serum. Helm et al.’s [25] method was referred to. Briefly, 96-well microtiter plates were coated with 5 μg/mL *β*-conglycinin in carbonate buffer (0.05 M, pH 9.6) and incubated at 4 °C overnight; then, appropriately diluted serum from each chicken was added. After incubation with HRP-conjugated rabbit anti-chicken IgY and IgM antibody (Beijing Biosynthesis Biotechnology Co., Ltd., Beijing, China), phenylenediamine was used to visualize antibody binding. Absorbance was measured at 492 nm; data are expressed in optical density (OD) units.

### 2.8. RNA Extraction and Real-Time PCR

RNA from intestinal mucosa (duodenum, jejunum, and ileum) samples from broiler chicks was isolated using the TRIZOL method, and the concentration and purity of RNA were determined by Nanodrop-one. According to the instructions of the PrimeScriptTM RT reagent Kit with gDNA Eraser (Takara Biotechnology, Dalian, China), the RNA was then reversely transcribed to cDNA. Real-time PCR using the TB Green^®^ Premix Ex Taq kits (Takara Biotechnology, Dalian, China) was performed on a StepOnePlus™ (AppliedBiosystems, Carlsbad, CA, USA) with a 20 μL total volume reaction; all experiments were performed in triplicate. Primers for the target genes were obtained from the NCBI official website. The level of mRNA relative expression was calculated using the 2^−ΔΔCt^ method after normalization with *β*-actin as a housekeeping gene [26]. The primer sequences utilized in this investigation are provided in Table 2.

### 2.9. Microbiota Community Analysis

The contents of the cecum, which was kept at −80 °C, were removed and placed in dry ice and sent for testing (Personal Biotechnology, Nanjing, China). Total microbial DNA was extracted from cecal digesta samples using a commercial stool DNA extraction kit (Omega Bio-Tek, Norcross, GA, USA) following the manufacturer’s instructions. The quantity and quality of extracted DNAs were measured using a NanoDrop NC2000 spectrophotometer (Thermo Fisher Scientific, Waltham, MA, USA) and agarose gel electrophoresis, respectively. The PCR amplification of the bacterial 16S rRNA genes’ V3-V4 region was performed using the forward primer 338F (5′-ACTCCTACGGGAGGCAGCA-3′) and the reverse primer 806R (5′-GGACTACHVGGGTWTCTAAT-3′). PCR amplicons were purified with Vazyme VAHTSTM DNA Clean Beads (Vazyme, Nanjing, China) and quantified using the Quant-iT PicoGreen dsDNA Assay Kit (Invitrogen, Carlsbad, CA, USA). After the individual quantification step, amplicons were pooled in equal amounts, and pair-end 2 × 250 bp sequencing was performed using the Illlumina NovaSeq platform with NovaSeq 6000 SP Reagent Kit (500 cycles) at Nanjing Personal Biotechnology Co., Ltd. (Nanjing, China). α-diversity and *β*-diversity were analyzed on the online platform of Personalbio Genescloud (https://www.genescloud.cn/, accessed on 2 January 2024.).

### 2.10. Statistical Analysis

Data analysis was performed using SPSS version 26.0 (IBM Corp, Armonk, NY, USA). Levene’s test and the Shapiro–Wilk test were performed to assess the homogeneity of variances and the normality of the response variables prior to statistical analysis. For normally distributed data, a one-way analysis of variance (ANOVA) and Duncan’s multiple range test were used to determine statistical significance, and linear and quadratic comparisons were applied to determine the dose–effect response of *β*-conglycinin in broilers. The data were presented as means, the standard error of the mean (SEM), and *p*-values, and all differences were judged significant at *p* < 0.05. Furthermore, a taxon-dependent analysis was conducted using the Greengenes2 database (version: 2022.10) [27]. The sequence length distribution was calculated, the visualization distribution of taxonomic components was performed using QIIME2 (version: 2023.3) [28], and the analysis results were presented as histograms. Alpha diversity indices (Chao 1 index, observed species index, Shannon index, Simpson index) were obtained through the QIIME 2 package. Differences in relative bacterial abundance (phylum and genus level) and α-diversity data were analyzed using a non-parametric Kruskal–Wallis sum-rank test and Duncan’s multiple range test. PCoA was visualized via the vegan R package (version: 2.5–7).

## 3. Results

### 3.1. Growth Performance

As presented in Table 3, the FBW, ADG, and FCR of the treatments were significantly lower than those of the control group (*p* < 0.05). Meanwhile, compared with the control group as well as the 1% group, the ADFI was significantly reduced in the 2%, 3%, 4%, and 5% groups (*p* < 0.05). Additionally, the FBW, ADG, ADFI, and FCR showed both linear and quadratic responses to the *β*-conglycinin supplementation levels (*p* < 0.05). However, there was no significant difference among these treatment groups in the FCR; the FBW, ADG, and ADFI were not significantly different between the 2%, 3%, 4%, and 5% groups (*p* > 0.05).

### 3.2. Nutrient Utilization and Digestive Enzymes

As indicated in Table 4, *β*-conglycinin supplementation levels had linearly and quadratically significant impacts on CP, EE, and CF utilization and had quadratically significant impacts on DM and AME utilization (*p* < 0.05). Compared with the control group, the utilization of DM, CP, CF, and AME was significantly reduced in the treatment groups (*p* < 0.05), while the utilization of CP, CF, and AME was insignificantly different between treatment groups (*p* > 0.05). Furthermore, EE utilization was significantly reduced in the 2%, 3%, 4%, and 5% groups compared to the control group and 1% group (*p* < 0.05). However, the experimental treatments had no linearly and quadratically discernible impacts on ash utilization (*p* > 0.05). The duodenal content of digestive enzymes diminished linearly and quadratically with increased dietary levels of *β*-conglycinin (*p* < 0.05). Contrasted with the control group and 1% group, dietary supplementation with 2%, 3%, 4%, and 5% *β*-conglycinin significantly reduced the content of chymotrypsin and lipase (*p* < 0.05). In addition, the amylase levels of the treatments were significantly lower than those of the control group, and treatments with 2%, 3%, 4%, and 5% *β*-conglycinin diets had the highest content of amylase (*p* < 0.05). However, the content of lipase and amylase had no significant differences observed among the 2%, 3%, 4%, and 5% treatments (*p* > 0.05).

### 3.3. Serum Histamine Levels and β-Conglycinin-Specific IgY and IgM Levels

As summarized in Table 5, the serum levels of histamine and *β*-conglycinin-specific IgY and IgM varied linearly and quadratically between the treatments. Upon the addition of *β*-conglycinin, compared to the control group, the serum histamine levels were rapidly increased and had a fluctuating trend of increasing first and then decreasing (*p* < 0.05). Notably, the 3% group exhibited the highest level of histamine. The serum levels of *β*-conglycinin-specific IgY and IgM in all treatments were also significantly higher than those of the control group (*p* < 0.05). Furthermore, the *β*-conglycinin-specific IgM levels of broilers fed a diet supplemented with 3%, 4%, and 5% *β*-conglycinin were higher than those of their counterparts receiving a level of 1% and 2% (*p* < 0.05), but there was no significant difference between the 3%, 4%, and 5% groups (*p* > 0.05).

### 3.4. Serum Cytokine Levels

**Figure 1:**
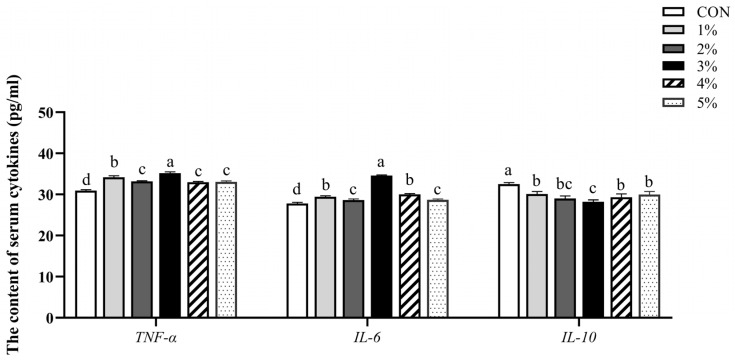
Effect of *β*-conglycinin on serum cytokine levels of broilers. TNF-α, tumor necrosis factor-alpha; IL-6, interleukin-6; IL-10, interleukin-10. ^a–d^ Value columns with different lowercase letters are statistically significantly different (*n* = 12; *p* < 0.05).

As displayed in Figure 1, compared to the control group, the serum levels of TNF-α and IL-6 were significantly increased and had a trend of increasing first and then decreasing, while the levels of IL-10 were significantly decreased and had a trend of decreasing first and then increasing (*p* < 0.05). Furthermore, we noticed that the levels of TNF-α and IL-6 were the highest, and the level of IL-10 was the lowest in the 3% group.

### 3.5. Tight Junction Protein Gene Expression in Duodenal, Jejunal, and Ileal Mucosa

**Figure 2:**
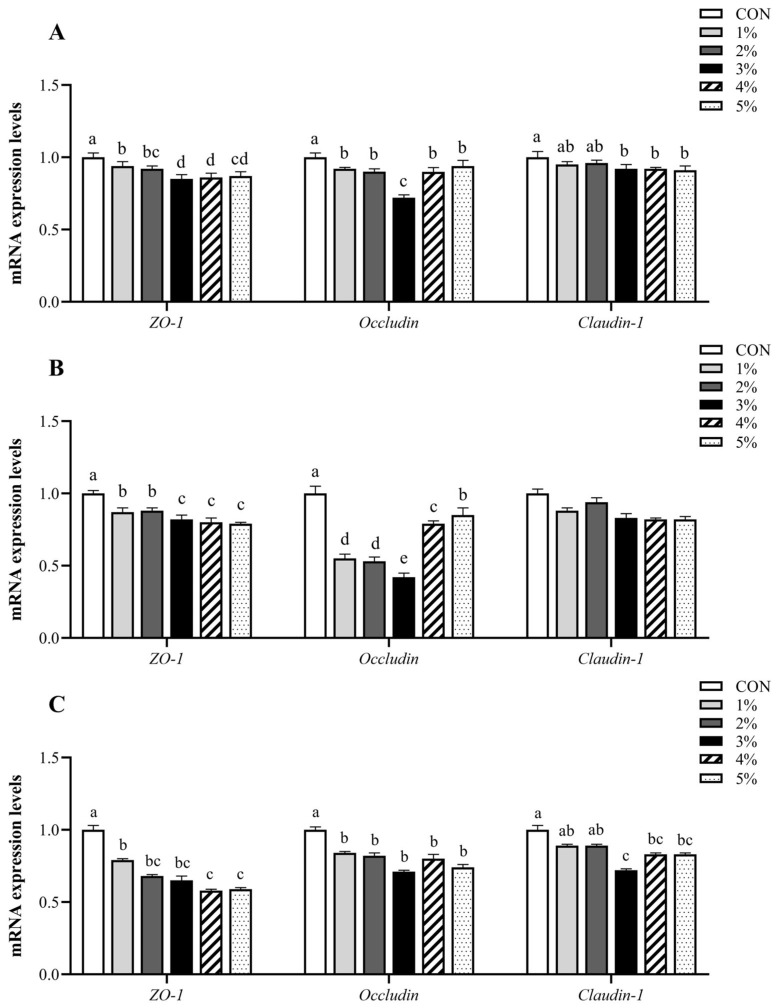
*ZO-1*, *Occludin*, and *Claudin-1* mRNA expression levels in the small intestinal mucosa. (**A**–**C**) present the mRNA expression levels of *ZO-1*, *Occludin*, and *Claudin-1* in the mucosa of the duodenum, jejunum, and ileum, respectively. *ZO-1*, Zonula Occludens-1. ^a–e^ Value columns with different lowercase letters are statistically significantly different (*n* = 12; *p* < 0.05).

As depicted in Figure 2, compared with the control group, the mRNA expression levels of *ZO-1* and *Occludin* in small intestinal mucosa were significantly down-regulated in treatment groups (*p* < 0.05). The mRNA expression levels of *Claudin-1* in duodenal and ileal mucosa were down-regulated considerably more in the 3%, 4%, and 5% groups than in the control group (*p* < 0.05); however, the *Claudin-1* expression levels in jejunal mucosa had no significant difference between groups (*p* > 0.05). Furthermore, the mRNA expression levels of *ZO-1* and *Claudin-1* in each intestinal mucosa were also not significantly different between the 3%, 4%, and 5% groups (*p* > 0.05). Notably, the dietary supplementation with 3% *β*-conglycinin resulted in lesser tight junction protein expression than other experimental groups.

### 3.6. Serum Levels of DAO and D-LA

According to the data presented in Table 6, *β*-conglycinin supplementation levels had linearly and quadratically significant impacts on DAO levels. In contrast to the control group, the serum levels of DAO were significantly increased in the treatments, and the levels in the 2% and 3% groups were higher than those of other treatment groups (*p* < 0.05). Nevertheless, dietary *β*-conglycinin inclusion had no linearly or quadratically significant effects on serum D-LA levels (*p* > 0.05).

### 3.7. MUC1 and MUC2 Gene Expression in the Duodenal Mucosa

**Figure 3:**
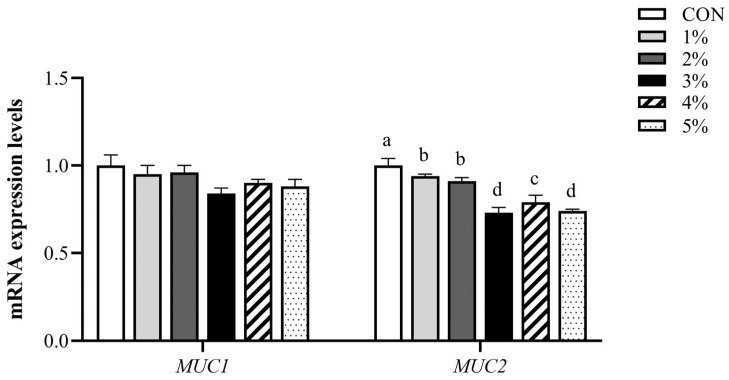
*MUC1* and *MUC2* mRNA expression levels in duodenal mucosa. *MUC1*, Mucin-1; *MUC2*, Mucin-2. ^a–d^ Value columns with different lowercase letters are statistically significantly different (n = 12; *p* < 0.05).

Figure 3 demonstrates that dietary inclusion with different levels of *β*-conglycinin significantly down-regulated the *MUC2* mRNA expression levels compared to the control group, and dietary supplementation with 3% and 5% showed lower expression levels (*p* < 0.05). However, no significant differences in *MUC1* mRNA expression levels were observed between groups (*p* > 0.05).

### 3.8. TNF-α, IL-6, and IL-10 Gene Expression in Duodenal, Jejunal, and Ileal Mucosa

**Figure 4:**
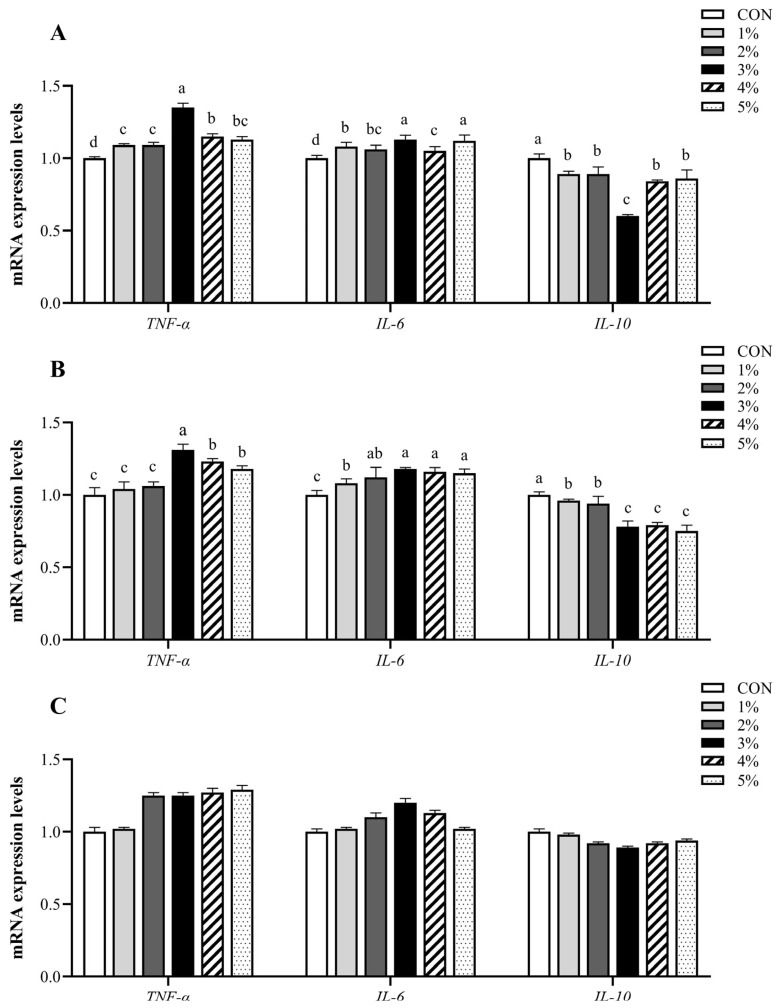
*TNF-α*, *IL-6*, and *IL-10* mRNA expression levels in the small intestinal mucosa. (**A**–**C**) represent the mRNA expression levels of *TNF-α*, *IL-6*, and *IL-10* in the mucosa of the duodenum, jejunum, and ileum, respectively. *TNF-α*, tumor necrosis factor-alpha; *IL-6*, interleukin-6; *IL-10*, interleukin-10. ^a–d^ Value columns with different lowercase letters are statistically significantly different (*n* = 12; *p* < 0.05).

As illustrated in Figure 4, compared to the control group, the treatments resulted in a significant up-regulation of *IL-6* and *TNF-α* mRNA expression levels in the duodenal mucosa, while *IL-10* expression was significantly down-regulated (*p* < 0.05). Moreover, the expression levels of *TNF-α* initially increased and subsequently decreased, reaching their peak in the 3% group. In contrast, the expression levels of *IL-10* initially decreased and then increased, reaching their nadir in the 3% group. In jejunal mucosa, compared with the control group as well as 1% and 2% *β*-conglycinin supplementation, the *TNF-α* expression levels were significantly up-regulated in the 3%, 4%, and 5% groups (*p* < 0.05). The mRNA expression levels of *IL-6* were gradually up-regulated compared to the control group (*p* < 0.05), with the 3% *β*-conglycinin group exhibiting the highest level of *IL-6* expression before attaining stability. Additionally, the mRNA expression levels of *IL-10* were gradually down-regulated in treatments and exhibited the lowest level in the 3% group before attaining stability (*p* < 0.05). However, *β*-conglycinin treatment groups had no significant effect on the mRNA expression of cytokines in ileal mucosa (*p* > 0.05).

### 3.9. Cecal Microbiota Characterization

**Figure 5:**
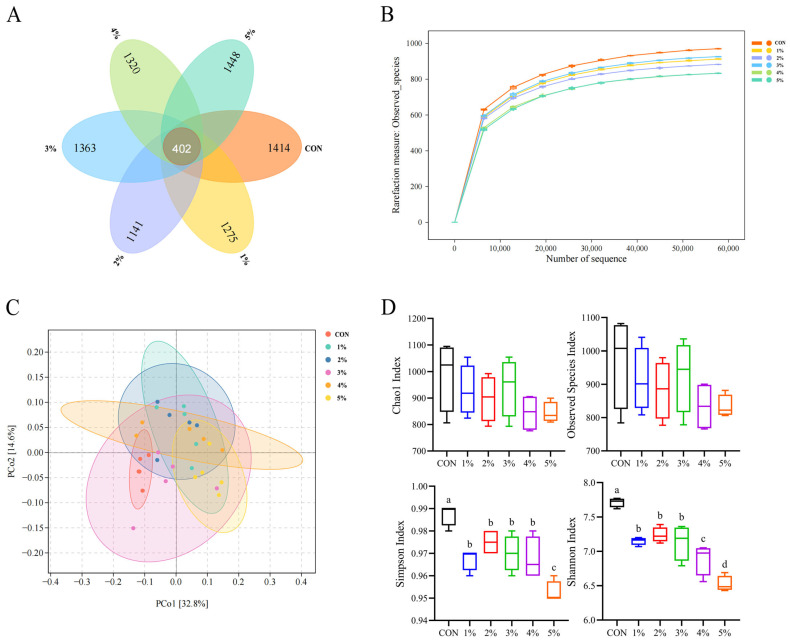

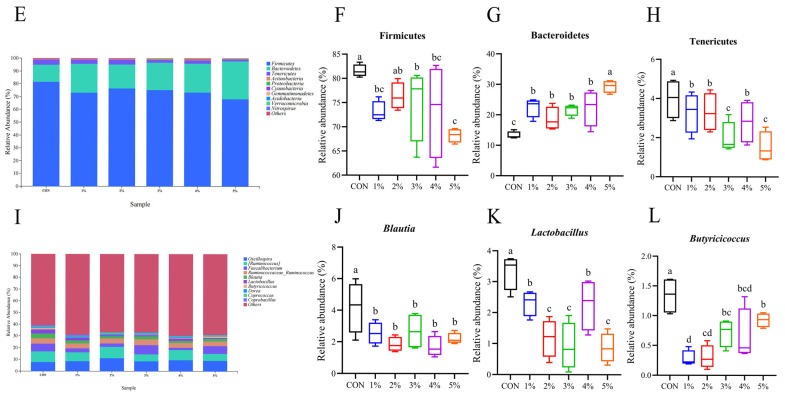
Effects of *β*-conglycinin on cecal flora composition of broilers. (**A**) Venn diagram of OTUs. (**B**) Rarefaction curves of samples. (**C**) Principal coordinate analysis of intestinal flora. (**D**) Alpha diversity analysis based on Chao 1, observed species, Simpson, and Shannon indices. (**E**) Cecal microbial composition at phylum level. (**F**–**H**) Differential cecal microbiota at phylum level. (**I**) Cecal microbial composition at genus level. (**J**–**L**) Differential cecal microbiota at genus level. In (**D**,**F**–**H**,**J**–**L**), the different colors (black/blue/red/green/purple/orange) were represented the control group, 1% group, 2% group, 3% group, 4% group, and 5% group, respectively. ^a–d^ Value columns with different lowercase letters are statistically significantly different (*n* = 12; *p* < 0.05).

The rarefaction curves of samples are presented in Figure 5B. As the number of sequencing exceeds 30,000, the curve flattens out, indicating that sufficient sequencing depth has been achieved and that the quality of this sequencing is credible.

There were 402 shared OTUs and 1414, 1275, 1141, 1363, 1320, and 1448 OTUs unique in the control, 1%, 2%, 3%, 4%, and 5% *β*-conglycinin groups, respectively. Venn diagrams showed that supplementation with *β*-conglycinin decreased the number of unique OTUs, meaning that *β*-conglycinin increased the percentage of shared bacteria (Figure 5A). As shown in Figure 5C, the results of the weighted UniFrac of principal coordinate analysis (PCoA) showed that the 1% and 5% treatment groups were clustered separately from the control group and had two principal coordinates (PCo1 and PCo2) explaining 32.8% and 14.6% of the variation, respectively. As presented in Figure 5D, dietary *β*-conglycinin addition significantly decreased the Shannon and Simpson indices (*p* < 0.05). However, the Chao1 and observed species indices had no special differences between groups (*p* > 0.05).

At the phylum level, as shown in Figure 5E–H, it was found that the intestinal dominant bacteria of the cecum of broilers were mainly composed of Firmicutes (74.62%), Bacteroidetes (21.31%), and Tenericutes (2.84%). The abundance of Firmicutes and Tenericutes was decreased considerably in the treatment group, while Bacteroidetes was increased (Supplementary Table S1). At the genus level, as shown in Figure 5I–L, Oscillospira (9.13%), Ruminococcus (7.77%), Faecalibacterium (4.78%), Ruminococcaceae unclassified (4.36%), Blautia (2.53%), Lactobacillus (1.81%), Butyricicoccus (0.70%), Dorea (0.69%), Coprococcus (0.64%), and Coprobacillus (0.57%) were dominant bacteria. Moreover, compared with the control group, the relative abundance of Blautia, Lactobacillus, and Butyricicoccus significantly decreased in the treatment group.

## 4. Discussion

Soybean is the most common source for production due to its nutritional and functional properties [29]. Unfortunately, *β*-conglycinin, which is a main storage protein, has long been identified as a potential antigenic and allergenic compound in soybean proteins [30]. It was reported that the ADG, ADFI, and FCR were all reduced when piglets received a diet containing 4% purified *β*-conglycinin [31]. Soybean lectin, as a heat-stable anti-nutritional factor like *β*-conglycinin, and a concentration of 0.24% of soybean lectin in the diet produced detrimental effects on chick nutrition [32]. Growth performance is an important indicator of the effectiveness of feeding. From the results of the present study, dietary *β*-conglycinin supplementation can decrease FBW, ADG, ADFI, and FCR in broilers. Meanwhile, we found that the dietary inclusion of *β*-conglycinin impaired the utilization of DM, CP, CF, EE, and AME in broilers. Several studies have demonstrated a significant positive correlation between growth performance and nutrient utilization in poultry [33,34]. Similarly, a previous study by Abdel-Raheem et al. [35] reported that DM, CP, and EE utilization was significantly lower in broilers fed soybean meal than those fed double-fermented soybean meal. Furthermore, in this study, *β*-conglycinin supplementation decreased chymotrypsin, amylase, and lipase in the duodenum, which is supported by the findings in other studies on fish [36]. The digestive enzymes in the intestine have been demonstrated to play a crucial role in the degradation of nutrients. Elevated levels of digestive enzymes in poultry may serve as an indicator of enhanced nutrient utilization and improved growth performance [37]. Therefore, these results indicate that dietary *β*-conglycinin inhibits the growth of broilers by reducing the synthesis and secretion of digestive enzymes and impairing nutrient utilization. Moreover, the growth performance of broilers is closely related to intestinal health [38,39]. We speculated that another reason for this was the possibility that *β*-conglycinin may trigger intestinal inflammation and compromise intestinal barrier function [40]. In other words, the observed negative impact on growth performance in this study can be attributed to *β*-conglycinin, which functions as an anti-nutritional factor that impedes digestion and absorption in broilers by compromising the integrity of the intestinal barrier.

Type I allergic reaction is an acute systemic allergic reaction due to the binding of receptors on mast cells to IgE, which causes complexes to accumulate on the cell surface and induces the degranulation of mast cells, eventually leading to the release of mediators [41]. Moreover, among the pre-formed and newly synthesized inflammatory substances released during mast cell degranulation, histamine remains the best characterized and most potent vasoactive mediator associated with the acute phase of type I allergic reaction [42]. It was reported that the release of histamine in animals was triggered in response to *β*-conglycinin sensitization [43,44]. In accordance with this observation, our current study revealed a significant elevation in histamine levels following the supplementation of *β*-conglycinin, with the highest level observed in the 3% group. Studies have found that Immunoglobulin Y (IgY) is widely distributed in amphibians, birds, and reptiles; is orthologous to mammalian IgG [45]; and has a similar ability to mediate allergic skin reactions as IgE [46]. Additionally, Immunoglobulin M (IgM) acts as the initial responder to foreign invaders, such as viral pathogens responsible for significant pandemics [47]. The present study demonstrated a significant increase in *β*-conglycinin-specific IgY and IgM levels in the serum of sensitized broilers by *β*-conglycinin. Li et al. [48] found that dietary 2–8% *β*-conglycinin significantly increased the level of *β*-conglycinin-specific antibody in the serum of Turbot. Thus, *β*-conglycinin exhibits the potential to be transported to both the local and systemic immune systems, thereby inducing sensitization in broilers and eliciting the production of histamine and serum-specific antibodies. Notably, severe allergic reactions were observed with 3% *β*-conglycinin supplementation.

It has been reported that allergic individuals stimulate mast cells to release mediators such as histamine and cytokines [49]. Cytokines are categorized into pro-inflammatory and anti-inflammatory cytokines, and the development of inflammation in the gut is accomplished by the collaboration of various types of pro-inflammatory and anti-inflammatory cytokines. It is well known that food protein-induced sensitization can manifest by high TNF-α expression in the small intestine and that TNF-α increases intestinal permeability in patients with inflammation [50]; IL-6 levels are correlated with the severity of inflammatory changes in the intestine [51]; and IL-10 is a key anti-inflammatory cytokine that ensures the protection of the host from overreactions to pathogens and microbiota while playing an important role in the treatment of enteritis, autoimmunity, cancer, and homeostasis in other environments [52]. In the present study, the levels of TNF-α and IL-6 significantly increased after dietary 1–5% *β*-conglycinin inclusion, while the levels of IL-10 significantly decreased. Studies have found that dietary 4% *β*-conglycinin significantly increased the serum levels of IL-6 in weaned piglets [53], Guo et al. [54] demonstrated that the oral administration of purified *β*-conglycinin to rats significantly increased TNF-α levels. The dietary addition of 6% *β*-conglycinin also secreted pro-inflammatory cytokines in hybrid grouper [55]. Furthermore, the observed changes in pro-inflammatory cytokine levels were consistent with the trends in serum histamine release, which reinforced the evidence that the *β*-conglycinin-triggered systemic immune-mediated inflammatory response triggered was indicative of allergic reactions. In particular, the addition of 3% *β*-conglycinin triggered a significantly more pronounced inflammatory response. In the present study, the dietary supplementation of *β*-conglycinin significantly up-regulated the *IL-6* and *TNF-α* mRNA expression levels in both the duodenal mucosa and jejunal mucosa of broilers while down-regulating the *IL-10* mRNA expression levels. This was similar to the results in mice [10], turbot [48], grass carp [56], and Chinese mitten crabs [57]. The disruption of the intestinal immune barrier is linked to the activation of intestinal immune cytokines [58]. During the occurrence of enteritis, the up-regulation of pro-inflammatory cytokines (including *IL-6* and *TNF-α*) acts as a protective inflammatory response and leads to inflammation response through the down-regulation of anti-inflammatory cytokines (including *IL-10*) [59]. Thus, the findings of this study indicated that *β*-conglycinin triggered an intestinal inflammatory response in broilers and induced damage to the immune barrier of the intestine. However, the impact of *β*-conglycinin on pro-inflammatory and anti-inflammatory cytokines in the ileal mucosa was not found to be significant, possibly attributed to a gradual decline in the immunoreactivity of *β*-conglycinin as it traverses through the alimentary canal [60]; therefore the immunoreactivity of *β*-conglycinin is progressively diminished in the ileum relative to the duodenum and jejunum. On the whole, *β*-conglycinin could damage intestinal immune barrier integrity by eliciting an inflammatory response in broilers.

The physical barrier is an intestinal epithelial structure composed of intestinal mucosal epithelial cells and the tight junctions between them and is the first line of defense between the intestinal lumen and the internal environment [61]. Tight junction (TJ) proteins are essential for maintaining this structure and include *Claudins*, *Occludins*, Zonula Occludens (*ZO*), and others [62]. The activity of DAO serves as a marker of intestinal permeability [63]. Previous studies have suggested that the levels of DAO increase in the broiler when the intestinal mucosa is injured [64]. Our studies have found that *β*-conglycinin significantly down-regulated *Occludin*, *Claudin*, and *ZO-1* expression levels in the intestine while significantly increasing DAO levels; this is consistent with the results for piglets and fish [13,65]. Therefore, our findings demonstrate that *β*-conglycinin may down-regulate the expression of tight junction proteins and enhance the permeability of the intestine, thereby compromising its physical barrier integrity in broilers. The intestinal chemical barrier is essential for the effective resistance and killing of pathogenic bacteria [66]. Among them, Mucoprotein (*MUC*) is crucial in maintaining the chemical barrier against infections and regulating innate immune responses to control inflammation [67]. In this study, dietary *β*-conglycinin supplementation significantly down-regulated the mRNA expression levels of *MUC2* in the intestine. Similar results were also observed by Wang et al. [13], who found that 6% *β*-conglycinin treatment induced a low mRNA expression level of *MUC2* in piglets. Therefore, the findings of this study suggest that *β*-conglycinin induces a certain degree of impairment to the chemical barrier of the intestine in broilers, further weakening the tight junction.

Interestingly, we further observed that low doses of *β*-conglycinin had a more pronounced impact on indicators such as serum histamine, cytokines, and intestinal gene expression in broilers compared to high doses of *β*-conglycinin. We hypothesized that broilers may demonstrate oral tolerance towards high doses of *β*-conglycinin due to established protective mechanisms. The induction of a low systemic immune response to soluble dietary proteins is known as oral tolerance, and oral tolerance has evolved to prevent allergic reactions to food proteins present in mucosal flora [68]. In addition, high doses of antigen induce clonal nonresponse or clonal deletion, which stimulates the body to exhibit an anisotropic nonresponse state when exposed to antigenic substances [69]. Similarly, piglets [7], mice [43], and mirror carp [15] also showed oral tolerance to high doses of *β*-conglycinin. We also observed that broilers demonstrate a higher sensitivity to *β*-conglycinin than piglets and fish due to the differences among species. Further investigation is warranted to elucidate the underlying mechanisms that generate tolerance.

The complex microbial composition of the chicken gastrointestinal tract is important for chicken health and performance, and the stability of the bacterial community maintains nutrient digestion and absorption and immune defense [70]. In the present study, the Simpson and Shannon indices were significantly decreased with the progressive addition of *β*-conglycinin. It is generally accepted that the high alpha diversity of the intestinal microbiota correlates with good host health [71]. Consequently, the decreased microbial diversity observed may be responsible for the disrupted intestinal health in birds fed *β*-conglycinin. PCoA showed that the treatment group of *β*-conglycinin was clustered separately from the control group, which means that this analysis truly represents the differences between the treatment group and the control group. At the phylum level, there was an observed reduction in Firmicutes and Tenericutes abundance after dietary *β*-conglycinin supplementation. Firmicutes could be associated with improved nutrient and energy utilization [72]; some species of Tenericutes play a crucial role in metabolizing recalcitrant carbon sources and safeguarding the host’s intestine against viral invasions [73,74]. Meanwhile, we found that the dietary inclusion of *β*-conglycinin increased the abundance of Bacteroidetes. This study showed that Bacteroidetes may act as pathogens to infect and harm the gastrointestinal tract [75]. Additionally, at the genus level, we found that supplemented *β*-conglycinin decreased the relative abundance of Blautia, Lactobacillus, and Butyricicoccus. Liu et al. [76] found a negative correlation between Blautia abundance and markers of obesity-related metabolic disorders. Butyricicoccus can promote growth performance, enhance immune response, and strengthen the intestinal barrier function of the host, and it also can produce butyric acid, which lowers intestinal pH and inhibits the proliferation of harmful bacteria [77]. Lactobacillus has positive effects on growth performance, nutrient utilization, and the immune system of animals [78,79]. Thus, the dietary inclusion of *β*-conglycinin may exert a detrimental impact on growth and metabolism in broilers. As discussed above, *β*-conglycinin reduced the relative abundance of beneficial bacteria and altered the delicate balance of the intestinal flora, thereby disrupting intestinal health in broilers. Notably, in this study, although the dietary inclusion of 1% *β*-conglycinin was demonstrated to have various negative effects on broilers, the effects of a dietary inclusion of less than 1% *β*-conglycinin on broilers still need to be further verified.

## 5. Conclusions

In conclusion, the aforementioned results substantiate our conjectures that the dietary supplementation of 1.0%, 2.0%, 3.0%, 4.0%, and 5.0% *β*-conglycinin induced allergic and inflammatory responses; compromised intestinal barrier integrity; and disrupted the balance of the intestinal microbiota, thereby exerting inhibitory effects on nutrient utilization and growth performance in broilers, with the 3% group demonstrating the most negative outcomes. Therefore, in the present study, *β*-conglycinin inclusion in the formula feed for broilers should not exceed a dose of 1%, i.e., the *β*-conglycinin content in the diet should not exceed 0.6% (converted from *β*-conglycinin purification purity).

## Figures and Tables

**Table 1:** The ingredients and nutrient level of experimental diets (DM basis, %).

Ingredients	*β*-Conglycinin Level (%)					
	CON	1%	2%	3%	4%	5%
*β*-conglycinin	0.00	1.00	2.00	3.00	4.00	5.00
Corn	62.51	62.55	62.29	62.12	61.87	62.22
Casein	3.95	3.27	2.58	1.60	0.88	0.10
Corn DDGS	10.00	10.00	10.00	10.00	10.00	10.00
Corn gluten meal	11.70	11.60	11.45	11.30	11.10	10.70
Rapeseed meal	4.70	4.66	4.67	4.72	4.74	4.69
Soybean oil	1.99	1.85	1.93	2.11	2.25	2.09
Limestone	1.43	1.42	1.41	1.39	1.37	1.35
Dicalcium phosphate	1.70	1.67	1.71	1.75	1.78	1.82
Salt	0.17	0.15	0.15	0.15	0.15	0.15
L-lysine HCL	0.83	0.82	0.81	0.82	0.82	0.82
DL-methionine	0.09	0.10	0.10	0.12	0.13	0.14
Threonine	0.18	0.17	0.17	0.17	0.16	0.16
Tryptophan	0.06	0.06	0.06	0.06	0.06	0.06
Valine	0.00	0.00	0.00	0.02	0.02	0.03
Vitamin–mineral premix ^1^	0.50	0.50	0.50	0.50	0.50	0.50
Nutrient levels ^2^						
Dry matter	88.14	88.19	88.28	88.37	88.46	88.51
Crude protein	20.50	20.71	20.89	20.87	21.00	21.00
Ether extract	5.13	4.98	5.03	5.18	5.28	5.12
Crude fiber	2.90	2.90	2.90	2.90	2.90	2.90
Apparent metabolizable energy (MJ/kg)	12.55	12.54	12.56	12.58	12.61	12.57
Calcium	1.01	1.00	1.00	1.00	1.00	1.00
Total phosphorus	0.69	0.68	0.68	0.68	0.68	0.68
Lysine	1.35	1.35	1.35	1.35	1.35	1.35
Methionine	0.54	0.54	0.54	0.54	0.54	0.54
Threonine	0.90	0.90	0.91	0.91	0.90	0.91
Tryptophan	0.22	0.22	0.22	0.22	0.22	0.22
Valine	1.01	1.01	1.01	1.01	1.01	1.01

^1^ The vitamin–mineral premix provided the following per kilogram of diet from 1 to 21 days of age (supplied per kilogram of feed): vitamin A, 4000 IU; vitamin D3, 1200 IU; vitamin E, 15 IU; vitamin K, 0.5 mg; vitamin B1, 2.8 mg; vitamin B2, 4.0 mg; vitamin B3, 35 mg; vitamin B5, 20 mg; vitamin B6, 3.5 mg; vitamin B12, 0.01 mg; biotin, 0.2 mg; folic acid, 0.6 mg; choline chloride, 1000 mg; Cu, 9 mg; Fe, 90 mg; Zn, 90 mg; Mn,80 mg; I, 0.25 mg; Se, 0.15 mg. ^2^ The nutrient levels were calculated values. The AME of *β*-conglycinin was calculated based on the determination of the nutrient content of *β*-conglycinin and a formula provided by Tian and Li (1995) [23]: AME(MJ/kg) = 5.49 + 0.12 × CP + 0.14 × EE.

**Table 2:** Primers used for real-time PCR.

Gene Name	Primer Sequences (5′ to 3′)	Product Size (bp)	Accession No.
*ZO-1*	F: TGGGCCTCACGGACTAAAAT R: GTTTGCTCCAACAAGATAGTTTGG	118	XM_413773.4
*Claudin-1*	F: TGATTGCTTCCAACCAGGCT R: CACACGGCTCTCCTTGTCTA	89	NM_001013611.2
*Occludin*	F: ATGCACCCACTGAGTGTTGG R: GAGGTGTGGGCCTTACACAG	93	NM_205128.1
*TNF-α*	F: GAGCGTTGACTTGGCTGTC R: AAGCAACAACCAGCTA TGCAC	176	NM_214022.1
*IL-6*	F: CGCCCAGAAATCCCTCCTC R: AGGCACTGAAACTCCTGGTC	203	NM_204628.1
*IL-10*	F: AGAAATCCCTCCTCGCCAAT R: AAATAGCGAACGGCCCTCA	121	NM_001004414.2
*MUC1*	F: GTGCCGACGAAAGAACTG R: TGCCAGGTTCGAGTAAGAG	187	XM_021089728.1
*MUC2*	F: CTGTGTGGGGCCTGACAA R: AGTGCTTGCAGTCGAACTCA	65	XM_021082584.1
*β*-actin	F: CACCACAGCCGAGAGAGAAA R: CACAGGACTCCATACCCAAGAA	215	NM_205518.2

Abbreviations: *ZO-1*, Zonula Occludens-1; *TNF-α*, tumor necrosis factor-alpha; *IL-6*, interleukin-6; *IL-10*, interleukin-10; *MUC1*, Mucin-1; *MUC2*, Mucin-2.

**Table 3:** The effects of *β*-conglycinin administration on the growth performance of broilers.

Items	*β*-Conglycinin Level (%)						SEM	*p*-Value		
	CON	1%	2%	3%	4%	5%		Treatment	Linear	Quadratic
IBW (g)	38.13	38.56	38.38	38.35	38.35	37.96	0.155	0.93	0.65	0.58
FBW (g)	682.79 ^a^	603.62 ^b^	536.46 ^c^	509.99 ^c^	511.96 ^c^	527.03 ^c^	13.376	<0.01	<0.01	<0.01
ADG (g/d)	30.70 ^a^	26.91 ^b^	23.72 ^c^	22.46 ^c^	22.55 ^c^	23.29 ^c^	0.718	<0.01	<0.01	<0.01
ADFI (g/d)	42.83 ^a^	41.08 ^a^	37.90 ^b^	35.92 ^b^	35.95 ^b^	36.02 ^b^	0.654	<0.01	<0.01	<0.01
FCR	1.40 ^a^	1.53 ^b^	1.61 ^b^	1.61 ^b^	1.59 ^b^	1.55 ^b^	0.019	<0.01	<0.05	<0.01

^a–c^ Means within each row with different superscripts are significantly different (*p* < 0.05). Data is presented as mean ± SEM (*n* = 12). Abbreviations: IBW, initial body weight; FBW, final body weight; ADG, average daily gain; ADFI, average daily feed intake; FCR, feed conversion ratio.

**Table 4:** The effects of *β*-conglycinin inclusion on the nutrient utilization and digestive enzymes of broilers.

Items	*β*-Conglycinin Level (%)						SEM	*p*-Value		
	CON	1%	2%	3%	4%	5%		Treatment	Linear	Quadratic
Nutrient utilization (%)										
DM	85.92 ^a^	83.46 ^b^	83.26 ^b^	80.79 ^c^	80.21 ^c^	80.83 ^c^	0.504	<0.01	0.66	<0.05
CP	78.75 ^a^	71.40 ^b^	71.09 ^b^	72.78 ^b^	71.99 ^b^	71.77 ^b^	0.682	<0.01	<0.05	<0.05
EE	88.07 ^a^	88.06 ^a^	83.79 ^b^	83.00 ^b^	79.63 ^c^	80.02 ^c^	0.782	<0.01	<0.01	<0.01
CF	69.64 ^a^	60.78 ^b^	60.39 ^b^	58.38 ^b^	61.74 ^b^	60.55 ^b^	0.858	<0.01	<0.01	<0.01
Ash	40.71	40.63	40.08	39.71	39.62	39.70	0.763	0.10	0.60	0.86
AME	87.69 ^a^	85.01 ^b^	85.10 ^b^	83.43 ^b^	83.39 ^b^	82.97 ^b^	0.441	<0.01	0.45	<0.05
Digestive enzymes (U/mg prot)										
Chymotrypsin	1.98 ^a^	1.93 ^a^	1.76 ^b^	1.72 ^b^	1.40 ^c^	1.50 ^c^	0.043	<0.01	<0.01	<0.01
Lipase	9.94 ^a^	9.54 ^a^	7.90 ^b^	7.55 ^b^	7.47 ^b^	7.23 ^b^	0.246	<0.01	<0.01	<0.01
Amylase	1.40 ^a^	1.33 ^b^	1.25 ^c^	1.21 ^c^	1.27 ^c^	1.24 ^c^	0.016	<0.01	<0.01	<0.01

^a–c^ Means within each row with different superscripts are significantly different (*p* < 0.05). Data is presented as mean ± SEM (*n* = 12). Abbreviations: DM, dry matter; CP, crude protein; CF, crude fiber; EE, ether extract; Ash, crude ash; AME, apparent metabolizable energy.

**Table 5:** Effects of *β*-conglycinin supplementation on serum histamine and *β*-conglycinin-specific IgY and IgM levels of broilers.

Items	*β*-Conglycinin Level (%)						SEM	*p*-Value		
	CON	1%	2%	3%	4%	5%		Treatment	Linear	Quadratic
Histamine (pg/mL)	27.34 ^d^	31.67 ^c^	31.03 ^c^	34.92 ^a^	32.38 ^b^	32.55 ^b^	0.431	<0.01	<0.01	<0.01
IgY (OD_492nm_)	0.64 ^d^	0.70 ^c^	0.71 ^bc^	0.76 ^a^	0.74 ^ab^	0.73 ^ab^	0.008	<0.01	<0.01	<0.01
IgM (OD_492nm_)	0.88 ^d^	0.95 ^c^	1.14 ^b^	1.26 ^a^	1.31 ^a^	1.29 ^a^	0.032	<0.01	<0.01	<0.01

^a–d^ Means within each row with different superscripts are significantly different (*p* < 0.05). Data is presented as mean ± SEM (*n* = 12). Abbreviations: IgY, Immunoglobulin Y; IgM, Immunoglobulin M.

**Table 6:** Effects of *β*-conglycinin administration on serum Diamine oxidase (DAO) and D-lactic acid (D-LA) levels of broilers.

Items	*β*-Conglycinin Level (%)						SEM	*p*-Value		
	CON	1%	2%	3%	4%	5%		Treatment	Linear	Quadratic
DAO (U/mL)	9.19 ^c^	13.38 ^b^	14.86 ^a^	14.75 ^a^	13.47 ^b^	12.68 ^b^	0.411	<0.01	<0.05	<0.01
D-LA (μmol/mL)	0.15	0.20	0.19	0.18	0.18	0.21	0.010	0.57	0.20	0.45

^a–c^ Means within each row with different superscripts are significantly different (*p* < 0.05). Data is presented as mean ± SEM (*n* = 12). Abbreviations: DAO, Diamine oxidase; D-La, D-lactic acid.

## Data Availability

Data are contained within this article.

## Supplementary Materials

The following supporting information can be downloaded at: https://www.mdpi.com/article/10.3390/ani15121701/s1, Table S1. Relative abundances of the dominant bacteria of broilers at phylum level (%).
