# Supplementary material for: Soybean β-Conglycinin Inhibits Broiler Growth and Nutrient Utilization by Inducing Allergic and Inflammatory Responses, Impairing Intestinal Barrier Integrity and Altering Cecal Microbiota
Source: Animals (Basel). 2025 Jun 9;15(12):1701. doi: 10.3390/ani15121701 (PMC12189050; doi:10.3390/ani15121701)
Supplement: Supplementary file 1 [file animals-15-01701-s001.zip › animals-3616921-supplementary.pdf]

Table S1. Relative abundances of the dominant bacteria of broilers at phylum level (%).

Item	β-conglycinin level (%)						SEM	*p*-value
	CON	1%	2%	3%	4%	5%		
Oscillospira	7.76	8.50	11.18	8.53	9.70	9.08	0.529	0.55
Ruminococcus	9.10	7.54	9.42	6.03	8.49	6.02	0.453	0.08
Faecalibacterium	6.36	3.45	2.98	7.63	1.83	6.43	0.855	0.30
Ruminococcaceae Ruminococcus	4.77	4.14	4.31	4.95	4.06	3.91	0.219	0.75
Blautia	4.19^{a}	2.54^{b}	1.84^{b}	2.67^{b}	1.70^{b}	2.21^{b}	0.240	<0.05
Lactobacillus	3.33^{a}	2.31^{b}	1.18^{c}	0.91^{c}	2.27^{b}	0.87^{c}	0.220	<0.05
Butyricicoccus	1.34^{a}	0.28^{d}	0.31^{cd}	0.72^{bc}	0.65^{bcd}	0.93^{b}	0.090	<0.05
Dorea	0.68	0.70	0.73	0.63	0.82	0.59	0.040	0.68
Coprococcus	0.77	0.70	0.85	0.63	0.46	0.43	0.052	0.09
Coprobacillus	0.84	0.93	0.45	0.48	0.32	0.43	0.073	0.07

$^{a\text{-}d}$ Means within each row with different superscripts are significantly different ($p < 0.05$). Data is presented in mean ± SEM (n = 12).
